# Epidemiological and Oral Public Health Aspects of Dental Pain: A Narrative Review

**DOI:** 10.7759/cureus.74908

**Published:** 2024-12-01

**Authors:** Víctor J Delgado-Pérez, Nuria Patiño-Marín, Vicente Rueda-Ibarra, Sonia Márquez-Rodríguez, Alejandro J Casanova-Rosado, Juan F Casanova-Rosado, Gladys R Acuña-González, Sandra A López-Gómez, Carlo E Medina-Solís, Gerardo Maupomé

**Affiliations:** 1 Doctoral Program in Dental Sciences, Faculty of Dentistry, Autonomous University of San Luis Potosí, San Luis Potosí, MEX; 2 Academic Area of Dentistry of Health Sciences Institute, Autonomous University of Hidalgo State, Pachuca, MEX; 3 Laboratory of Clinical Research, Faculty of Stomatology, Autonomous University of San Luis Potosí, San Luis Potosí, MEX; 4 School of Dentistry, Autonomous University of Campeche, Campeche, MEX; 5 Advanced Studies and Research Center in Dentistry "Dr. Keisaburo Miyata" School of Dentistry, Autonomous University of State of Mexico, Toluca, MEX; 6 Department of Epidemiology, Richard M. Fairbanks School of Public Health, Indiana University, Indianapolis, USA

**Keywords:** dental pain, oral epidemiology, oral health, oral public health, risk factors

## Abstract

The aim of this narrative review is to synthesize and discuss existing evidence on the epidemiological aspects of dental pain, addressing its prevalence, risk factors, population distribution, impact on the quality of life, and implications for public health. Dental pain is a common condition that involves complex mechanisms of pain transmission and perception. Dental pain can be due to various causes, such as caries, pulpitis, periodontitis, dental trauma, and soft tissue conditions (e.g., stomatitis). In addition, psychological and social factors influence pain perception and severity, such as anxiety and previous negative experiences. The global burden of dental pain is considerable, generating a significant impact on the quality of life of people at individual and collective levels. This impact manifests itself in a reduction in productivity, due to absenteeism from work and school, as well as difficulties in performing daily tasks. In addition, dental pain entails high costs for health systems, including expenses associated with diagnosis, treatment, and the care of complications arising from the lack of timely care. These costs also include indirect expenses associated with the loss of productivity and impacts on the general well-being of the population. The magnitude of these effects, both at individual and population levels, underlines the importance of prevention and access to timely and effective treatment of dental pain.

The prevalence of dental pain varies by region and factors such as age, socioeconomic status, and access to services. Risk factors include sociodemographic (age, gender, and ethnicity), socioeconomic (income, education, and occupation), psychological (anxiety and depression), and behavioral (oral hygiene and diet) dimensions. People with lower socioeconomic and educational status are more likely to suffer from dental pain due to a lack of access to services and inadequate preventive practices. Dental pain negatively affects the quality of life, disrupting daily functions and causing emotional distress. Public health proposes improving access to preventive dental care, oral health education, and equitable policies to reduce disparities. Strategies aimed at reducing the burden of dental pain at the population level include expanding access to dental care, promoting healthy habits, and ameliorating the significance of care costs as a barrier. Future research should focus on innovative assessment methods, expanding the accessibility to various levels of care, and understanding the biopsychosocial determinants of dental pain to develop effective interventions.

## Introduction and background

Dental pain is a global public health problem that affects people's well-being, health systems, and society's productive processes. The widespread occurrence and multifactorial etiology of this event require a comprehensive approach to effectively manage its burden and promote equitable access to oral healthcare. The experience of dental pain is more than just a sensation; it involves biological, psychological, and social factors that affect the onset, severity, and impact on daily life [1]. The biological basis of dental pain, which may arise from a variety of sources, such as infectious (dental caries, periodontitis, and abscesses), traumatic (fractured teeth and temporomandibular joint dysfunction), or other intraoral pathological processes (pulpitis, gingivitis, and stomatitis), has been well-established. The underlying mechanisms include complex processes related to pain transmission and perception, often following nerve injury or the inflammation of the tissues involved [2,3]. However, these biological factors cannot explain the differences in pain we see across populations.

Psychosocial components are equally important in determining how a specific individual perceives dental pain. Previous pain conditions, in conditions of anxiety and depression, also often increase pain perception and worsen the quality of life when poorly treated through drug therapy programs. Furthermore, previous negative experiences with dental treatment can also lead to the avoidance of dental care and worsened oral health status due to anxiety caused by dental treatment [4,5]. Dental pain is also significantly affected by social determinants including (but not limited to) socioeconomic status, the access and affordability of dental care, cultural beliefs about dental care or providers, and the health literacy of individuals residing in specific communities. Individuals in lower socioeconomic groups generally have more difficulty obtaining timely and appropriate dental care, resulting in the postponement of treatment for worse conditions and more acute and prolonged pain attacks [6].

Untreated or poorly managed dental pain has global implications. Direct costs encompass those related to the diagnosis, treatment, and management of complications. Indirect costs are enormous due to lost days of work and school, reduced cognitive ability, and decreased social relationships. It can have a major effect on the overall quality of life, influencing not only physical functioning and well-being but also mental health and social participation. Overall, the increased awareness of these complex biological, psychological, and social determinants can guide actions to help minimize the burden caused by dental pain and improve equity in oral health across different populations [7,8]. The International Association for the Study of Pain (IASP) emphasizes that dental pain can be influenced by personal experiences such as mechanical hypersensitivity, as well as psychological and social factors [9,10].

The objective of this narrative review is to synthesize and discuss existing evidence on the epidemiological aspects of dental pain, addressing its prevalence, risk factors, population distribution, impact on the quality of life, and implications for public health.

## Review

Search methodology

The present narrative review on the "epidemiological and public health aspects of dental pain" employed a search strategy to identify relevant evidence. The literature search was conducted in the PubMed and Google Scholar databases, two repositories widely recognized for their coverage of scientific literature in the health field. The selection of these databases is justified by their broad international reach and the quality of the indexed information. To maximize the sensitivity of the search, a strategy was used that integrated a variety of search terms, including synonyms and Medical Subject Headings (MeSH) terms in the case of PubMed. The descriptors used were a combination of keywords, such as "dental pain", "toothache", "odontalgia", "orofacial pain of odontogenic origin", "epidemiology", "risk factors", "prevalence", "incidence", "quality of life", "health inequalities", and "public health interventions". Different combinations of these terms were applied using Boolean operators (AND, OR, and NOT) to refine the search and obtain relevant results.

The inclusion and exclusion criteria were established to ensure the quality and relevance of the selected articles. The review was limited to studies published in the last 10 years, in order to include only updated evidence and reflect the most recent advances in the field. Priority was given to epidemiological studies with a cross-sectional, longitudinal, and ecological design, including meta-analyses and systematic reviews with peer review that specifically analyzed the epidemiological and public health aspects of dental pain in human populations. The exclusion criteria included studies not directly related to the epidemiology of dental pain, studies in animals or in vitro models, publications in languages ​​other than English or Spanish, studies that were not peer-reviewed, studies with poor methodologies or with a high risk of bias, and any article that did not fit the defined time frame. A complementary manual search was also carried out in the reference lists of key articles identified during the initial database searches. This allowed us to identify relevant studies that might not have been retrieved through electronic searches.

Finally, the identified articles were independently evaluated by two reviewers. Once the eligible articles were selected, a narrative analysis of the extracted information was carried out, with the aim of describing, synthesizing, and contextualizing the evidence on the epidemiological and public health aspects of dental pain.

Global burden of dental pain

Good oral health allows individuals to eat, speak, smile, and socialize without discomfort, pain, or shame and is considered both subjective and dynamic. An individual's ability to adapt to physiological changes over their lifetime and maintain their own teeth and mouth through independent self-care reflects good oral health [11,12]. The increasing prevalence of oral diseases raises concerns in many low- and middle-income countries due to broader social, economic, and commercial challenges. These diseases are a global public health issue [13,14]. Oral diseases can have substantial effects, such as pain, sepsis, reduced quality of life, lost school days, family disintegration, decreased labor productivity, and high dental treatment costs for individuals, families, and health systems. Their economic burden encompasses direct costs (treatment expenses), indirect costs (productivity losses due to work and school absenteeism), and intangible costs (such as pain and issues with biting, chewing, eating, tasting, speaking, and expressing emotions such as smiling, all related to social and family activities) [14]. In 2015, dental diseases accounted for US$356.8 billion (US$476.32 billion in November 2024) in direct costs and US$187.61 billion (US$250.45 billion in November 2024) in indirect costs worldwide, totaling worldwide costs due to dental diseases of US$544.41 billion (US$726.78 billion in November 2024) [15].

Studying the epidemiology of dental pain is crucial to gain insights into its prevalence, distribution, and determinants. This understanding is essential for developing effective public health interventions and treatment strategies [6,16,17]. Kumarswamy emphasizes that dental pain can be caused by several stimuli, including bacterial infections, enamel erosion, and gingival recession [18]. Some authors highlight the need for advancements in pain assessment methods, considering the aging global population [7,19,20].

The global burden of dental pain is substantial, with prevalence rates varying across different regions and populations worldwide. Additionally, factors such as untreated dental caries, the perceived need for dental treatment, and the impact on the quality of life contribute to an increased likelihood of experiencing dental pain. Understanding these regional variations and associated factors is crucial for developing targeted interventions to address dental pain in specific populations [14]. In a review of the literature conducted on children and adolescents, Slade found that the prevalence of dental pain ranged from 5% to 33%, increasing with the child's age, the severity of caries, and decreasing socioeconomic status [21]. More recently, it has been observed that the lifetime prevalence of dental pain among children and adolescents varies significantly across countries. Thus, two systematic reviews that included both groups reported overall prevalence rates of dental pain reaching 36.2% (95% CI = 33.0-39.4) [22] in one review and 32.7% (95% CI = 29.6-35.9) [23] in the other. The differences in rates may lie in the methods used to assess pain or pertain to different age groups, participant characteristics, regions, and public policies; specifically, studies differ in the time frames used to inquire about dental pain experiences (most commonly three, six, or 12 months) [24]. Many children in low- and middle-income countries and indigenous populations in high-income countries have reported a history of dental pain throughout their lives. Many adults have limited access to dental care, which means that they also have to cope with both acute and chronic dental pain and reduced quality of life [14].

Risk factors for dental pain

Various risk factors contribute to the development of dental pain, encompassing sociodemographic, socioeconomic, psychological, and behavioral factors. Additionally, some authors discuss the challenges in assessing acute dental pain and emphasize the need to address ethical issues, data bias, and evidence collection for the practical implementation of these technologies in clinical dentistry. This knowledge underscores the multifaceted nature of the risk factors for dental pain and the need for comprehensive approaches to address them [7,17].

Sociodemographic factors

Sociodemographic determinants, such as age, gender, and ethnicity, are crucial to understanding the epidemiology of dental pain and its relationship with oral conditions in general. These factors not only influence the biological vulnerability of individuals but also play an essential role in the social determinants of health, which include socioeconomic context, access to healthcare, and cultural practices related to oral hygiene [21-29]. Age is a key determinant; for example, older children may experience a higher prevalence of caries [30-32] and, as a consequence, dental pain due to a lack of adequate oral hygiene and competition in accessing dental treatments [1,21,23,33,34]. In contrast, among older adults, the occurrence of dental pain may be related to the presence of periodontal diseases, systemic diseases, and the use of medications that affect oral health [35,36]. Sex also affects the perception and management of dental pain. Research has shown that women may report higher levels of dental pain and greater discomfort, which may be related to differences in pain care, health expectations, and perceptions [22,25,34]. Regarding ethnicity, disparities in access to dental services and cultural differences in pain perception and treatment may lead to significant variations in the prevalence and severity of dental pain between different groups [25,34].

Therefore, addressing the epidemiology of dental pain requires public health strategies that not only take into account these individual determinants but also integrate a holistic approach that addresses health disparities. The implementation of effective preventive interventions must consider biological vulnerabilities along with social and economic barriers faced by diverse population groups.

Socioeconomic factors

Socioeconomic factors are well-established determinants of the occurrence of dental pain and play a fundamental role in the prevalence and severity of oral health conditions [21,34,37-40]. These factors encompass a wide range of dimensions, including income [22,38], education [22,38,40], occupation [17,34], and access to healthcare services [21,34]. They shape the experience of dental pain by affecting individuals' ability to maintain oral health, seek timely treatment, and adopt preventive behaviors [17,22,41].

One of the most direct socioeconomic determinants of dental pain is income level. Individuals from lower-income households are disproportionately affected by dental pain due to reduced access to dental care services, higher treatment costs, and limited insurance coverage [17,25,34,38,41]. Studies show that financial constraints are a significant barrier to receiving dental care, leading to untreated or delayed oral health issues that may exacerbate pain. Individuals with lower incomes report a higher frequency of dental pain, often due to untreated caries and periodontal conditions [34,42,43].

Educational level is another key factor influencing oral health outcomes and pain perception [34,38]. Individuals with lower educational levels are less likely to be informed about preventive oral health practices and may have a limited understanding of the need for regular dental visits [11,44]. Low health literacy has been linked to poor oral hygiene habits, a higher risk of oral diseases, and increased rates of dental pain. Research has demonstrated that populations with lower educational levels exhibit higher rates of untreated caries, significantly contributing to their experience of dental pain [45-47].

Employment status and occupational categories also influence dental pain experiences. Individuals with low-paying and highly stressful jobs, or those experiencing job insecurity, are more likely to neglect their dental health due to time constraints, the lack of dental benefits, or conflicting financial priorities [11,17,48]. Furthermore, individuals without dental insurance are less likely to seek preventive care and more likely to present advanced painful oral diseases [14,21,49,50].

Psychological factors

Psychological factors associated with dental pain refer to the emotional and cognitive aspects that influence the perception, intensity, and management of dental pain. The experience of pain is not merely a physical response to a harmful stimulus but is modulated by mental interpretation, emotional state, and patient expectations. Therefore, dental pain may be perceived as more severe or tolerable depending on various psychological factors that, in many cases, amplify the perception of discomfort or contribute to its relief [51-53]. Dental anxiety is one of the most prevalent emotional responses to dental pain. It refers to intense fear or apprehension toward dental treatment, which can lead to the avoidance of appointments and worsened oral health. Dental anxiety is associated with an amplified perception of pain and negative past experiences that increase patient sensitivity [54,55]. Depression is another relevant psychological factor that can influence the experience of dental pain. Patients with depression tend to have a lower pain tolerance and a more negative perception of their overall health, which may lead them to perceive even mild dental pain as unbearable or difficult to manage [56-58]. Chronic stress affects pain perception through the sustained activation of the hypothalamic-pituitary-adrenal axis and the sympathetic nervous system response. This not only contributes to hypersensitivity in the nervous system but also can lead to harmful dental health habits, such as teeth grinding or jaw clenching, increasing the incidence of dental pain [56,59,60]. Additionally, psychological factors such as fear may exacerbate the perception of dental pain, while protective psychosocial factors such as a sense of coherence, social support, and self-esteem can modulate pain perception and facilitate coping with risk events [61-63].

Behavioral factors

Behavioral factors play an important role in the development of dental pain, with oral hygiene practices and dietary habits being key aspects to consider [64-66]. Research has shown that cariogenic diets, poor oral hygiene, and low fluoride exposure are associated with dental caries, the commonest biological cause of dental pain [67,68]. Furthermore, it has been found that attitudes toward oral hygiene and the self-care of teeth correlate with both recent negative experiences in dental practices and negative childhood experiences, highlighting the influence of past dental experiences on current oral hygiene behavior. Understanding these behavioral factors is crucial for developing effective preventive strategies and intervention programs to address dental pain [69-71].

Impact of dental pain on the quality of life

The impact of dental pain on the quality of life of affected individuals extends beyond physical discomfort and encompasses functional and psychosocial consequences. Acute pain from hard tissues in dentistry can result from infectious and inflammatory dental diseases, trauma-related cases, and personal experiences such as mechanical hypersensitivity [72-74]. Additionally, fear and dental anxiety are common and can exacerbate pain sensitivity, influencing patients' subjective experiences during dental procedures [75-77]. Evaluating the impact of oral disorders on health-related quality of life involves several approaches, including constructing scales to indicate the extent of functional and psychosocial consequences, assessing patients' perceptions of events, and ranking functional disorders and their social consequences in a hierarchy of outcomes [78-82]. These approaches, exemplified by measurements such as the General Oral Health Assessment Index (GOHAI) [81-83], the Oral Health Impact Profile (OHIP) [72,78,79], the CPQ 11-14 [80], the ECOHIS [80], and the B-ECOHIS [80], aim to capture the frequency and severity of oral health issues and their effects on functional and psychosocial well-being. Understanding the multifaceted impact of dental pain on the quality of life is crucial for developing comprehensive strategies that address the holistic burden of this condition. National surveys on oral health-related quality of life conducted in Western Europe, Australia, and the United States show that dental conditions contribute to lower life satisfaction. In adults, orofacial pain is common and is a major contributing factor to decreased quality of life worldwide [14,84].

Dental pain and oral public health implications

Dental pain represents a significant public health issue due to its widespread prevalence and its impact on individual well-being, healthcare systems, and social productivity. Untreated dental pain can be a manifestation of more severe oral diseases, such as infections, abscesses, and tooth loss, disproportionately affecting vulnerable populations with limited access to dental care. This pain often reduces the quality of life, affecting daily activities such as eating, speaking, and sleeping, and is a leading cause of school and work absenteeism [35,72,78-80,84]. From a public health perspective, dental pain highlights the need for preventive and accessible oral healthcare services and education, particularly in underserved communities. Strategies such as improving access to routine dental care and promoting oral health literacy can lessen the burden of dental pain while ultimately improving population health outcomes and reducing healthcare costs [11,85,86].

Addressing the socioeconomic determinants of dental pain requires comprehensive public health strategies focused on reducing disparities in access to care and promoting equitable oral health. Policies aimed at expanding dental insurance coverage, enhancing oral health literacy, and offering targeted preventive programs for low-income and underserved populations are essential for mitigating the impact of these socioeconomic factors on dental pain. A limited number of studies have provided evidence of the considerable social cost of oral diseases in terms of negative effects on employment status and labor productivity [14].

Public policies aimed at reducing the burden of dental pain

Several proposed public policies designed to reduce the burden of dental pain have prioritized prevention, access to care, and equity in oral health services. Key strategies are mentioned below.

Universal Access to Preventive Care

Expanding access to regular dental checkups, fluoride treatments, and sealants through public healthcare programs is crucial for preventing the onset of dental pain and its associated complications, especially for low-income and vulnerable populations [11,87,88].

Oral Health Education

National campaigns aimed at improving oral hygiene practices, promoting the use of fluoride toothpaste, and encouraging healthy dietary habits can reduce the prevalence of dental diseases that cause pain [89-91].

Integration of Oral Health Into Primary Care

Training primary care providers to identify and treat early signs of oral health issues can help reduce the incidence of untreated dental pain, particularly in regions with a shortage of dental professionals [92,93].

Affordable Dental Care Policies

National and local governments can implement subsidies or insurance coverage for essential dental treatments for vulnerable groups, ensuring that cost is not a barrier to controlling dental pain [94,95].

Focus on Social Determinants of Health

Addressing broader determinants such as education, socioeconomic status, access to clean water, and nutrition can reduce disparities in oral health outcomes and lower the prevalence of dental pain in disadvantaged communities [96,97].

The implementation of these policies has the potential to significantly mitigate the long-term dental pain outlook at both the individual and societal levels. By promoting better oral health in the population, there would be not only a decrease in the incidence of dental pathologies and associated complications but also a reduction in the economic and logistical burden these diseases impose on healthcare systems. This, in turn, would contribute to a more efficient use of healthcare resources, alleviating pressure on emergency services and allowing for a more preventive rather than reactive approach in dental care. Additionally, improved oral health positively impacts the quality of life, reducing absenteeism in the workplace and schools and enhancing the overall well-being of the community.

## Conclusions

The epidemiological aspects of dental pain present a complex interaction of biological, psychological, and social factors. Future research on the epidemiological aspects of dental pain should continue to explore these multifaceted dimensions while addressing ethical considerations and developing innovative assessment methods to enhance clinical dental practices. Public health proposes improving access to preventive dental care, oral health education, and equitable policies to reduce disparities, together with removing the distal cause of poor oral health, such as inordinate access to sweetened sugar beverages and the multiple sources of simple carbohydrates that exist in many societies. Strategies aimed at reducing the burden of dental pain at the population level include expanding access to dental checkups, promoting healthy habits, and ensuring that cost is not a barrier.
